# Microbial community structural response to variations in physicochemical features of different aquifers

**DOI:** 10.3389/fmicb.2023.1025964

**Published:** 2023-02-09

**Authors:** Heng Dai, Yiyu Zhang, Wen Fang, Juan Liu, Jun Hong, Chaowang Zou, Jin Zhang

**Affiliations:** ^1^State Key Laboratory of Biogeology and Environmental Geology, China University of Geosciences, Wuhan, China; ^2^School of Environmental Studies, Hubei Key Laboratory of Yangtze Catchment Environmental Aquatic Science, China University of Geosciences, Wuhan, China; ^3^School of Environmental Studies, China University of Geosciences, Wuhan, China; ^4^Hubei Shuili Hydro Power Reconnaissance Design Institute, Wuhan, China; ^5^State Key Laboratory of Hydrology-Water Resources and Hydraulic Engineering, Yangtze Institute for Conservation and Development, Hohai University, Nanjing, China; ^6^Chinese Academy of Sciences, Xinjiang Institute of Ecology and Geography, Ürümqi, China

**Keywords:** groundwater, microbial communities, aquifer, chemical-physical analyses, multiple locations

## Abstract

**Introduction:**

The community structure of groundwater microorganisms has a significant impact on groundwater quality. However, the relationships between the microbial communities and environmental variables in groundwater of different recharge and disturbance types are not fully understood.

**Methods:**

In this study, measurements of groundwater physicochemical parameters and 16S rDNA high-throughput sequencing technology were used to assess the interactions between hydrogeochemical conditions and microbial diversity in Longkou coastal aquifer (LK), Cele arid zone aquifer (CL), and Wuhan riverside hyporheic zone aquifer (WH). Redundancy analysis indicated that the primary chemical parameters affecting the microbial community composition were NO_3_^–^, Cl^–^, and HCO_3_^–^.

**Results:**

The species and quantity of microorganisms in the river–groundwater interaction area were considerably higher than those in areas with high salinity [Shannon: WH (6.28) > LK (4.11) > CL (3.96); Chao1: WH (4,868) > CL (1510) > LK (1,222)]. Molecular ecological network analysis demonstrated that the change in microbial interactions caused by evaporation was less than that caused by seawater invasion under high-salinity conditions [(nodes, links): LK (71,192) > CL (51,198)], whereas the scale and nodes of the microbial network were greatly expanded under low-salinity conditions [(nodes, links): WH (279,694)]. Microbial community analysis revealed that distinct differences existed in the classification levels of the different dominant microorganism species in the three aquifers.

**Discussion:**

Environmental physical and chemical conditions selected the dominant species according to microbial functions. *Gallionellaceae*, which is associated with iron oxidation, dominated in the arid zones, while *Rhodocyclaceae*, which is related to denitrification, led in the coastal zones, and *Desulfurivibrio*, which is related to sulfur conversion, prevailed in the hyporheic zones. Therefore, dominant local bacterial communities can be used as indicators of local environmental conditions.

## 1. Introduction

Groundwater is an important source and reservoir of freshwater across the world, accounting for 33% of the world’s total freshwater (de Graaf et al., 2019; Gleeson et al., 2020; Luijendijk et al., 2020). Compared with surface water, groundwater is less disturbed by external factors and can ensure a continuous and stable supply of good-quality fresh water over long time periods (Aeschbach-Hertig and Gleeson, 2012; Griebler and Avramov, 2015). However, human reliance on groundwater is increasing, and human activities are gradually affecting the quality of groundwater and ecological balance (Danielopol et al., 2003; Gerth and Förstner, 2004; Li et al., 2018; Ren and Zhang, 2020; Zhou B. et al., 2021; Lan et al., 2022).

Physicochemical indicators of groundwater can be used to measure water suitability for various types of production and domestic uses (Shahid et al., 2014; Vadiati et al., 2016; Dippong et al., 2019; Seben et al., 2022; Stylianoudaki et al., 2022; Wang et al., 2022). However, unique and regional hydrogeological features can be heterogeneous in their spatial distribution (Hernàndez-Diaz et al., 2019; Klingler et al., 2021; Labat et al., 2021). The groundwaters of different areas have different origins, and groundwater physicochemical parameters are closely related to aquifer type, such as loess (Zhou J. et al., 2021), karst (Ryu et al., 2020), and bedrock (Schulze-Makuch and Kennedy, 2000; Hynek et al., 2022). Differences in the climate and geological formations of the aforementioned aquifer types lead to variabilities in the water cycle and water balance. Annual rainfall as well as the moisture cycle pathways of humid areas are different from arid areas (Humphries et al., 2011; Huang et al., 2017). During circulation processes, marked differences exist in the degree of groundwater recharge and discharge in various regions, resulting in diverse gradients in the physicochemical features of the flow pathways (Yang et al., 2018; Unnikrishnan et al., 2021). Therefore, to protect groundwater, it is important to identify the main physicochemical indicators that affect water quality.

Groundwater systems are very sensitive to environmental changes, and the composition and distribution of their microbial communities are related to biogeochemistry (Grenthe et al., 1992; Clilverd et al., 2011; Romanak et al., 2012; Yuan W. et al., 2020). Clear correlations have been reported between changes in groundwater microbial communities and ion composition and salinity (Chandler et al., 2021). The composition and structure of microbial communities tend to evolve based on variations in ion concentration (Amalfitano et al., 2014; Sang et al., 2018; Chen et al., 2020, 2022; Chakraborty et al., 2022). Thus, each aquifer system has unique microbial communities. Studying the effects of physicochemical parameters on groundwater microbes can provide an in-depth understanding of differences in the geographical distribution of groundwater. At the same time, the microbial community and its functions are influenced by the groundwater ecological environment. As the main inhabitants of groundwater, microorganisms play major roles in key biogeochemical processes (Goldscheider et al., 2006; Merino et al., 2022). As the basis for the formation of groundwater ecosystems, microbial communities serve as important vehicles in groundwater that drive biogeochemical cycles, and the composition of microbial communities and their structure impact groundwater quality and ecological processes (Penny et al., 2003; Mouser et al., 2010; Amalfitano et al., 2014; Lu et al., 2022; Shi et al., 2022; Yang et al., 2022). Therefore, insightful studies of groundwater composition and microorganisms can contribute to the sustainable management and protection of groundwater resources.

Aquifers in different geographical locations show differences in their groundwater chemical composition, as many studies have reported. However, the mechanisms and degree to which specific chemical and physical water properties affect the dominant microorganisms and microbial interaction networks are not fully understood. The main purpose of this study was to investigate the driving forces behind water quality change in geographical environments and their impact on groundwater ecology. The groundwater of three typical aquifers, the Longkou coastal aquifer (LK), Cele arid area aquifer (CL), and Wuhan riverside hyporheic zone aquifer (WH) were chosen as the research objects. Based on 16S rDNA high-throughput sequencing, the characteristics of microbial community structural diversity in the groundwater of the three study areas and the response of microorganisms to environmental factors were analyzed. The differences in microbial structural diversity and functional characteristics in the different study areas were compared. The following topics were addressed: (1) the spatial distribution characteristics of groundwater quality in the specific aquifers; (2) factors affecting the physical and chemical properties of groundwater; and (3) evaluation of the response of microbial communities to the various structures of the aquifers. This study provides important reference information for assisting groundwater protection strategies.

## 2. Materials and methods

### 2.1. Study areas

**FIGURE 1:**
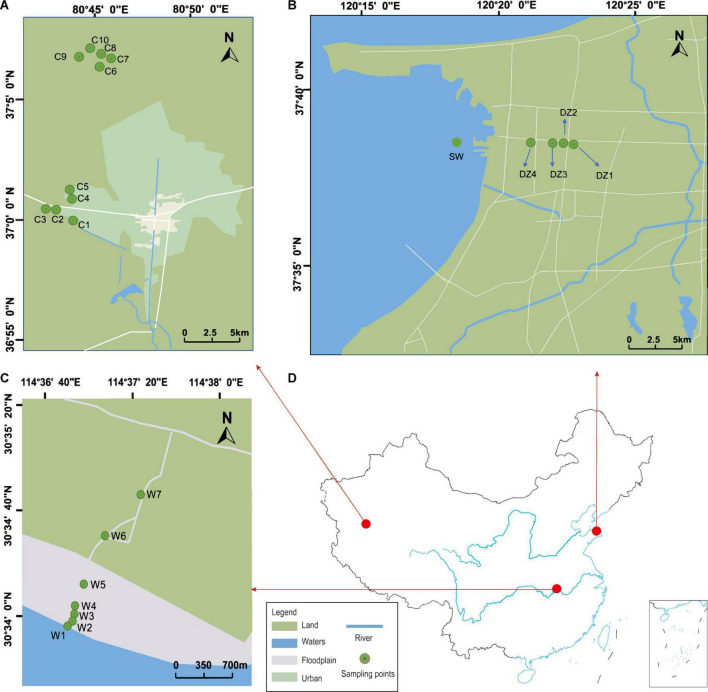
**(A)** Cele County is located in the northern part of the Kunlun Mountains, and “C1” to “C10” represent the 10 groundwater sampling sites. **(B)** Longkou city is located in the northwestern part of the Jiaodong Peninsula, China, and “DZ1” to “DZ4” represent the groundwater sampling points. **(C)** Wuhan city is located in the middle reaches of the Yangtze River, and “W1” represents river water samples and “W2” to “W7” represent groundwater sampling sites. **(D)** Map of China, with the red dots representing the locations of the sampling areas in China. The relevant latitude and longitude are indicated on the map for each sampling area.

A total of three study areas are involved in this study. They are geographically distant and have different sources of recharge. Thus, there is no groundwater exchange among the regions. Because aquifers in different geographical locations have different types and degrees of disturbances, they are typically subject to different environmental influences (Akhtar et al., 2020). As shown in Figure 1, Cele County (CL), Xinjiang Uygur Autonomous Region, is a typical plains oasis city, and most of the surrounding area is desert (Zhang et al., 2001). The surface water mainly originates from the Kunlun Mountains and is mainly recharged by snowmelt and precipitation. The general climate is characterized by intense sunlight, scarce precipitation, and strong evaporation, with an average annual precipitation of only 35.5 mm but an average annual evaporation of 2,751.6 mm (Cao T. et al., 2020). Longkou city, Shandong Province, is a typical coastal zone that is bordered by the Bohai Sea (Miao et al., 2018). The source of groundwater recharge is mainly atmospheric precipitation and surface water infiltration, with an average annual precipitation of 634.3 mm. Most of the city’s water comes from groundwater, which is strongly affected by seawater intrusion (Li et al., 2020). Wuhan city, Hubei Province, is a typical riverine city, and the Yangtze River flows through the city (Feng et al., 2007). The Yangtze River and its largest tributary, the Han River, meet in the city. In general, the three locations consist of an arid zone, a seawater intrusion zone, and a hyporheic zone.

### 2.2. Sampling method

Water samples were stored on-site in sterile 6 L plastic containers, and microorganisms were extracted onto sterile nitrocellulose filter membranes as quickly as possible, placed in sterile centrifuge tubes and stored on dry ice for subsequent microbiological analysis. At the same time, water samples were collected in triplicate using sterile 500 mL containers for physicochemical analyses in the laboratory. Some of the water chemistry parameters, including temperature, pH, dissolved oxygen (DO), electrical conductivity (EC), oxidation-reduction potential (ORP), and total dissolved solids (TDS), were measured and recorded at the field site using a portable multiparameter water quality analyzer (HANNA Instruments HI9829, Italy). The HCO_3_^–^ concentration was determined using an alkalinity kit (Merck, Germany), anions (Cl^–^, SO_4_^–^, and NO_3_^–^) were determined using an ion chromatograph (ICS-2500, USA), and cations (K^+^, Ca^2+^, Na^+^, and Mg^2+^) were tested using ion chromatography inductively coupled plasma emission spectroscopy (OPTIMA2000DV, USA). The accuracy of the tests was within ±5‰. The total organic carbon (TOC) values were determined by a TOC analyzer (Multi N/C-2100 TOC, Analytik Jena AG, Germany).

In Longkou city (Figure 1B), along the direction perpendicular to the coastal zone, water samples were collected at three different depths from the DZ1–DZ3 monitoring wells, only one set of shallow groundwater samples was collected from DZ4, and another set of seawater samples was collected on the west coast for comparison, for a total of eleven sets of water samples. In two areas of Cele County (Figure 1A), wells C1–C5 in the southern part of Cele and C6–C10 in the northwestern part of Cele were sampled. Among them, C2, C3, and C5 were shallow wells with water depths of 15–20 m, while the rest were deep wells with water depths of 60–80 m. A total of 10 sets of water samples were collected. At the Yangluo River bank in Wuhan (Figure 1C), along the Yangtze River in the vertical direction, W1 was the river water sampling point, W2, W3, W4, and W5 were the sampling points at 3–4 m deep monitoring wells on the river bank at locations 10, 50, 100 and 300 m from the river, respectively, W6 and W7 were the two monitoring points in a farmland area along the Yangtze River. Seven sets of water samples were collected in total. Three biological replicates were used subsequently for processing the water samples collected from the above areas.

### 2.3. Microbial sequencing

All microorganisms were extracted from 6 L of water using a vacuum filtration device that allowed the sample water to pass through a sterile 0.22 μm nitrocellulose membrane. The total DNA from the microorganisms was then extracted using a PowerSoil^®^ DNA Isolation Kit (Karczewski et al., 2017) and stored at −80°C in an ultralow temperature freezer until sequencing (Zhang et al., 2018). A polymerase chain reaction (PCR) was then performed on the V4 region of the bacterial gene using the 341F (5′CCTAYGGGRBGCASCAG-3′) and 806 R (5′-GGACATCNNGGGTACTA AT-3) primers (Sundberg et al., 2013). An ABI GeneAmp^^®^^ 9700 was used as the PCR instrument (Ramakers et al., 2003), and the reaction procedure was as follows: predenaturation at 59°C for 5 min, cyclic phase denaturation at 95°C for 30 s, followed by heat treatment at 55°C for 30 s, amplification at 72°C for 45 s, and finally denaturation at 72°C for 10 min until the 10°C stop. PCR products from all samples were pooled together for sequencing (PE) on an Illumina Hiseq2500 platform. The PE reads from sequencing were subjected to a series of splicing, quality control, and filtering procedures. In these processes, the splicing of gene sequence is completed, low quality sequences filtered to reduce the interference to downstream data analysis and obtain optimized sequences, which were clustered at 97% similarity for operational taxonomic units (OTU) clustering. Remove OTUs with a total abundance of 1 to reduce the impact of low abundance on subsequent analysis (Eren et al., 2013). Then, using the reference sequences from the Silva database (version number 138), (Quast et al., 2013) the RDP naïve Bayesian classifier was used to identify the species of the OTUs (Cole et al.’s 2009), and the taxonomic information corresponding to each OTU was obtained for the subsequent analysis of microbial composition and diversity.

### 2.4. Statistical analysis

Four representative indices (Shannon, Chao1, coverage, and Heip) for the alpha diversity of microbial communities were calculated using Mothur (1.30.2) to evaluate microbial diversity and richness, and the OTU similarity level used for index assessment was 97% (Chappidi et al., 2019). Qiime was used to calculate the beta diversity distance matrix (Caporaso et al., 2010), and then non-metric multidimensional scaling (NMDS) analysis and mapping using the package “vegan” in R were carried out to understand the degree of aggregation of different aquifer communities from an intuitive visual perspective. Redundancy analysis was used to investigate the relationship between sampling characteristics and microbial communities and to identify the main environmental factors affecting the distribution structure of microbial communities (Blackwood et al., 2003). Differences in species abundance between groups were detected based on linear discriminant analysis effect (LEfSe) (Segata et al., 2011), namely, LDA effect size analysis, which is an analysis tool for biological indicators of high-dimensional data. It can compare multiple groups, focus on statistical significance and biological correlations, and find biological indicators that have significant differences between groups. The R (version 3.3.1) tool was used to generate community histograms to determine the microbial composition of different groups (or samples) at each taxonomic level (domain, kingdom, phylum, order, family, genus, species, OTU, etc.) based on the results of taxonomic analysis. The original molecular ecological network was generated using molecular ecological nework analysis (MENA) (Deng et al., 2012) and illustrated with Cytoscape, which was used to show the relationships between microorganisms in the same geographical environment and the differences in microbial communities between different aquifers.

## 3. Results

### 3.1. Hydrogeochemical properties

#### 3.1.1. Physicochemical characteristics of groundwater in dissimilar sampling areas

The physicochemical indicators measured in the field and laboratory are listed in Tables 1–3. The data were analyzed by temperature, pH, and ORP.

**TABLE 1:** Location and environmental factors at the time of sampling in Longkou.

Station ID	Geographic coordinate	T (°C)	pH	TOC (mg/L)	EC (μs/cm)	TDS (g/L)	ORP (mv)	DO (mg/L)
DZ1-1	120°22′31′′ 37°38′28′′	16.5	7.62	1.7	1,366	0.683	13.4	4.93
DZ1-2	120°22′31′′ 37°38′28′′	16.9	7.58	2.1	1,018	0.515	22.0	1.72
DZ1-3	120°22′31′′ 37°38′28′′	15.7	7.66	2.9	794	0.397	14.1	3.92
DZ2-1	120°22′09′′ 37°38′29′′	17.0	7.30	1.8	1,816	0.910	20.5	1.38
DZ2-2	120°22′09′′ 37°38′29′′	17.1	7.49	3.9	1,302	0.651	13.2	1.54
DZ2-3	120°22′09′′ 37°38′29′′	16.3	7.72	1.0	682	0.341	14.1	3.01
DZ3-1	120°21′49′′ 37°38′30′′	16.6	7.35	1.7	682	1.262	5.7	4.40
DZ3-2	120°21′49′′ 37°38′30′′	15.6	7.32	1.1	2,443	1.223	16.2	2.09
DZ3-3	120°21′49′′ 37°38′30′′	17.3	7.33	1.5	2,076	1.040	14.3	2.64
DZ4-1	120°21′02′′ 37°38′30′′	17.2	7.07	2.3	1,866	0.933	19.1	1.71
SW	120°25′18′′ 37°37′20′′	14.2	8.02	6.1	4,224	21.130	25.8	4.74

TOC, total organic carbon; EC, electrical conductivity; TDS, total dissolved solids; ORP, oxidation-reduction potential; DO, dissolved oxygen.

**TABLE 2:** Location and environmental factors at the time of sampling in Cele.

Station ID	Geographic coordinate	T (°C)	pH	TOC (mg/L)	EC (μs/cm)	TDS (g/L)	ORP (mv)	DO (mg/L)
C1	80°43′53′′ 36°59′58′′	14.5	7.89	3.5	1,299	0.651	15.1	–
C2	80°42′59′′ 37°00′25′′	16.5	7.83	3.5	2,514	0.635	−55.1	–
C3	80°42′29′′ 37°00′27′′	19.8	7.82	5.1	2,314	0.501	−78.5	–
C4	80°43′49′′ 37°00′52′′	14.9	7.57	9.6	2,156	0.980	3.3	–
C5	80°43′40′′ 37°01′15′′	16.2	7.78	5.5	2,908	1.451	−55.3	–
C6	80°45′18′′ 37°06′22′′	19.0	7.78	0.3	2,635	1.259	−28.9	–
C7	80°45′52′′ 37°06′43′′	18.6	7.94	7.2	3,271	1.634	−146.2	–
C8	80°45′20′′ 37°06′55′′	18.2	7.97	5.3	3,998	1.997	−116.8	–
C9	80°44′10′′ 37°06′48′′	17.7	7.96	5.3	3,958	1.979	−124.5	–
C10	80°44′47′′ 37°07′09′′	17.0	7.97	4.4	6,712	3.354	−119.6	–

TOC, total organic carbon; EC, electrical conductivity; TDS, total dissolved solids; ORP, oxidation-reduction potential; DO, dissolved oxygen.

**TABLE 3:** Location and environmental factors at the time of sampling in Wuhan.

Station ID	Geographic coordinate	T (°C)	pH	TOC (mg/L)	EC (μs/cm)	TDS (g/L)	ORP (mv)	DO (mg/L)
W1	114°36′51′′ 30°33′57′′	10.5	8.72	2.1	425	0.213	−22.0	8.0
W2	114°36′58′′ 30°34′03′′	2.6	8.51	2.5	330	0.165	6.1	9.5
W3	114°36′59′′ 30°34′04′′	11.8	7.93	2.6	672	0.336	2.7	3.6
W4	114°37′01′′ 30°34′07′′	13.9	8.09	2.8	728	0.364	4.4	3.7
W5	114°37′03′′ 30°34′15′′	9.6	7.98	3.0	791	0.369	98.7	4.1
W6	114°37′07′′ 30°34′31′′	14.3	7.34	2.4	596	0.298	−71.1	3.1
W7	114°37′24′′ 30°34′46′′	11.0	7.53	6.3	730	0.365	−145.1	1.4

TOC, total organic carbon; EC, electrical conductivity; TDS, total dissolved solids; ORP, oxidation-reduction potential; DO, dissolved oxygen.

The temperature range in the coastal areas is 15.6–17.3°C, that in the arid areas is 14.5–19.8°C, and that in the hyporheic areas is 9.6–14.3°C. The temperature difference is small. Due to the different sampling seasons and depths, the groundwater temperature in the hyporheic areas is slightly lower.

The pH range in the coastal areas is 7.07–7.72, that in the arid areas is 7.57–7.97, and that in the hyporheic areas is 7.34–8.51. The fluctuation range of pH is in the order of hyporheic areas >coastal areas >arid areas.

In the coastal zone, except in DZ3-1, the vast majority of ORP values are between 13.2 and 22 mV, indicating that the groundwater is consistently in an oxidized state; in the arid zone, the distribution of ORP values changes from an average of −34.1 mV in the south to an average of −107.2 mV in the north, signifying an enhanced reduction state during groundwater flow. In the hyporheic zone, ORP changes from −145.1 mV to a maximum of 98.7 mV as the water gradually approaches the riverbank and then recovers to nearly 0 mV, reflecting a complex redox scenario at this location. The TDS criterion of less than 1 g/L is met in the coastal zone (0.341–1.262 g/L) except at DZ3; in the arid zone, the sampling points C1–C5 in the southern region meet the requirement, while C6–C10 in the northern region exceeds the criterion. In the hyporheic zone (0.165–0.396 g/L), all points meet the standard. The electrical conductivity in all three areas shows a pattern in which it increases with decreasing disturbance.

Except at W7, the sampling points in the coastal zone and hyporheic zone are within the TOC limit of 5 mg/L, whereas in the arid zone, all points exceed the standard except for C5, which meets the requirement. Accordingly, the temperature and pH of the three sites are not very different, but the ORP showed a unique local pattern, reflecting dynamic changes in the groundwater. The TDS standard is met at most of the sampling sites, but the values are relatively high in the northern part of the arid zone, and the conductivity is also slightly higher in the arid zone.

#### 3.1.2. Groundwater ion fluctuations in various sampling areas

**FIGURE 2:**
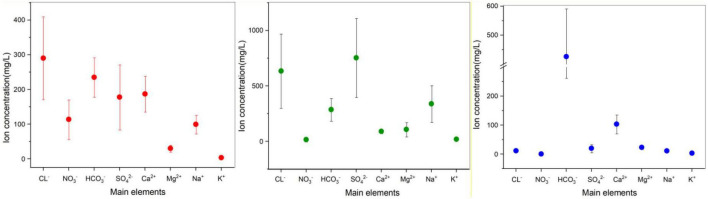
95% confidence interval error bar for the analysis of ion composition and fluctuations, divided into Longkou (red), Cele (green), and Wuhan (blue). The data points represent the mean value of each ion concentration, and the extension represents the confidence interval. Note that the seawater sample for the Longkou Formation is not included; its chemical composition varied substantially. Therefore, only the groundwater sample is considered here.

Based on the ion concentrations shown in Figure 2 and Tables 4–6, in each aquifer, each ion concentration was calculated with a 95% confidence interval to show its fluctuation, and the analysis was performed by different regional aquifers. The cations in the coastal areas are mainly Ca^2+^ (average concentration 187 mg/L, confidence interval 135–239 mg/L), followed by Na^+^ (average concentration 99 mg/L, confidence interval 72–126 mg/L). The anions are dominated by Cl^–^ (average concentration 290 mg/L, confidence interval 171–409 mg/L), followed by HCO_3_^–^ (average concentration 235 mg/L, confidence interval 178–291 mg/L). The main ions account for 71.4% of the total ions. By distance from the coast (Table 4), the ion concentrations vary as follows: K^+^ (2–3 mg/L) and Mg^2+^ (19–34 mg/L) change little, and Ca^2+^ (198–284 mg/L) and Na^+^ (81–174 mg/L) increase significantly. Cl^–^ (172–604 mg/L) increases dramatically, NO_3_^–^ (282–153 mg/L) decreases, and SO_4_^2–^ (114–166 mg/L) and HCO_3_^–^ (246–376 mg/L) increase slightly. These results show that the seawater intrusion process increases the proportion of groundwater ions such as Na^+^, Ca^2+^, and Cl^–^, and these gradually become the main ions, among which the concentration of Cl^–^ fluctuates the most, and the concentration of Na^+^ fluctuates less.

**TABLE 4:** The main elements in the Longkou water samples.

Station ID	Cl^–^ (mg/L)	NO_3_^–^ (mg/L)	HCO_3_^–^ (mg/L)	SO_4_^2–^ (mg/L)	Ca^2+^ (mg/L)	Mg^2+^ (mg/L)	Na^+^ (mg/L)	K^+^ (mg/L)
DZ1-1	172.79	282.53	246	114.10	198.16	19.15	81.05	2.02
DZ1-2	165.46	143.43	145	43.86	136.62	22.22	54.70	2.94
DZ1-3	120.29	119.97	176	39.30	83.71	22.10	67.36	2.98
DZ2-1	312.12	97.80	289	178.28	207.95	25.95	105.85	3.23
DZ2-2	264.68	23.29	209	149.04	149.40	23.79	105.00	2.35
DZ2-3	113.36	14.68	124	69.03	80.42	15.69	54.88	2.70
DZ3-1	604.33	153.16	376	166.65	284.20	34.71	174.42	3.36
DZ3-2	526.89	78.39	235	249.23	275.47	47.36	116.31	3.32
DZ3-3	350.97	168.20	223	319.86	229.63	60.51	96.26	4.31
DZ4-1	271.81	57.19	328	450.17	225.07	29.60	138.13	6.89
SW	16,281.80	31.20	17	2,571.29	366.84	1,036.42	8,591.48	383.72

**TABLE 5:** The main elements in the Cele water samples.

Station ID	Cl^–^ (mg/L)	NO_3_^–^ (mg/L)	HCO_3_^–^ (mg/L)	SO_4_^2–^ (mg/L)	Ca^2+^ (mg/L)	Mg^2+^ (mg/L)	Na^+^ (mg/L)	K^+^ (mg/L)
C1	163.68	18.54	128.14	229.15	86.61	32.95	161.31	10.14
C2	266.62	3.55	170.86	351.72	91.39	42.01	130.10	11.17
C3	163.87	0.87	170.86	276.02	89.55	31.02	89.49	10.37
C4	386.79	46.50	384.43	539.39	135.35	72.21	199.48	14.18
C5	554.43	75.80	543.08	734.83	127.10	120.35	289.35	24.43
C6	593.39	7.01	128.14	569.75	74.61	94.18	271.99	17.87
C7	663.21	2.77	286.79	799.93	47.60	99.97	406.48	22.31
C8	900.59	1.86	329.51	1,091.62	79.66	119.01	506.18	22.78
C9	951.99	1.90	268.49	1,069.45	93.18	121.82	489.74	24.35
C10	1,702.05	1.18	457.65	1,875.16	78.44	344.78	846.06	36.08

**TABLE 6:** The main elements in the Wuhan water samples.

Station ID	Cl^–^ (mg/L)	NO_3_^–^ (mg/L)	HCO_3_^–^ (mg/L)	SO_4_^2–^ (mg/L)	Ca^2+^ (mg/L)	Mg^2+^ (mg/L)	Na^+^ (mg/L)	K^+^ (mg/L)
W1	17.25	3.48	225.77	30.15	61.52	16.19	15.42	3.06
W2	15.16	<0.05	195.26	31.08	48.81	11.57	12.83	2.24
W3	9.52	0.16	360.02	3.86	111.17	26.66	13.71	4.01
W4	9.89	<0.05	622.40	4.96	134.18	30.73	9.68	4.09
W5	10.98	<0.05	494.26	39.61	130.49	25.42	6.92	3.31
W6	9.44	0.13	439.34	26.63	105.36	22.73	10.26	2.35
W7	8.33	0.12	646.81	3.60	131.51	29.46	8.67	3.45

In the arid zone from south to north (Table 5), the cations are dominated by Na^+^ (average concentration 339 mg/L, confidence interval 174–503 mg/L). The anions are dominated by SO_4_^2–^ (average concentration 753 mg/L, confidence interval 399–1,109 mg/L), followed by Cl^–^ (average concentration 634 mg/L, confidence interval 301–968 mg/L). The main ions account for 76.8% of the total ions. The ion concentrations vary as follows: K^+^ (14–24 mg/L) changes little, Ca^2+^ (105–74 mg/L) decreases slightly, and Na^+^ (173–504 mg/L) and Mg^2+^ (59–155 mg/L) increase significantly. Cl^–^ (307–962 mg/L) and SO_4_^2–^ (426–1,081 mg/L) increase dramatically, NO_3_^–^ (29–2.9 mg/L) decrease, and HCO_3_^–^ (279–294 mg/L) do not change much. The ion concentration is generally higher in the north than in the south. The main anions SO_4_^2–^ and Cl^–^ fluctuate substantially and to a similar extent, and the fluctuations are higher than that of the main cation Na^+^.

In the hyporheic zone (Table 6), the cations mainly consist of Ca^2+^ (average concentration 110 mg/L, confidence interval 76–144 mg/L), and the anions mainly consist of HCO_3_^–^ (average concentration 459 mg/L, confidence interval 282–637 mg/L). The proportion of main ions to the total ions is 89.5%. The ion concentrations vary as follows in order of proximity to the riverbank: K^+^ (3–4 mg/L), Mg^2+^ (29–26 mg/L), Ca^2+^ (131–111 mg/L), and Na^+^ (8–13 mg/L) all very little. Cl^–^ (8–9 mg/L) and NO_3_^–^ (0.12–0.16 mg/L) do not change significantly, SO_4_^2–^ (3.6 mg/L – 3 mg/L) fluctuates greatly, and HCO_3_^–^ (646–360 mg/L) decreases significantly. This shows that the cations do not change much and that the anions SO_4_^2–^ and HCO_3_^–^ participate in geochemical reactions that show drastic changes.

Overall, groundwater ion concentrations show various degrees of change due to diverse local disturbance factors, which results in an increase in the proportion of ions occupied, an overall increase in ion concentration, and a low ion concentration due to ion depletion.

#### 3.1.3. Significant relationships between major water chemical properties

The Pearson correlation analysis between the main physicochemical properties of the water samples can be seen in Supplementary Tables 1–3. In the coastal zone (Supplementary Table 1), TDS in the groundwater was significantly correlated with the major cations Ca^2+^, Na^+^, and Mg^2+^ and significantly correlated with the major anions Cl^–^, SO_4_^2–^, and HCO_3_^–^. The highest correlation coefficients were found between TDS and Ca^2+^ and Cl^–^, while the majority of groundwater samples were Ca-Cl type waters, except at DZ4-1 (Ca-SO_4_ type water). In the arid zone (Supplementary Table 2), TDS in the groundwater was significantly correlated with the major cations K^+^, Na^+^, and Mg^2+^ and significantly correlated with the major anions Cl^–^ and SO_4_^2–^. The highest correlation coefficients were those for TDS and Na^+^ with Cl^–^ and SO_4_^2–^, while the vast majority of groundwater samples were Na-Cl- or Na-SO_4_-type waters, except at C3 (Ca-SO_4_-type water). In the hyporheic zone (Supplementary Table 3), TDS in the groundwater was significantly correlated with the major cations Ca^2+^ and Mg^2+^ and significantly correlated with the major anion HCO_3_^–^. The samples were all Ca-HCO_3–_^–^type waters. A strong correlation between TDS and major ions was observed at all sites, but the dominant ions differed from place to place due to spatial distribution.

In analyzing the correlations, it was found that many ions have complex mutual correlations with each other. In the coastal zone (Supplementary Table 1), Ca^2+^, Na^+^, HCO_3_^–^, and Cl^–^ were significantly correlated with each other, indicating that these four types of ions have similar sources in the study area. In contrast, two ions, K^+^ and Ca^2+^, showed significant correlations with SO_4_^2–^, presumably due to the local dissolution of gypsum (CaSO_4_⋅2H_2_O) (Bischoff et al., 1994). In the arid zone (Supplementary Table 2), K^+^, Na^+^, Mg^2+^, Cl^–^, and SO_4_^2–^ were significantly correlated with each other, indicating that these five types of ions have similar sources in the study area. Ca^2+^ and NO_3_^–^ showed significant correlations, presumably mainly from the fertilization of agricultural fields and woods (Kavdir et al., 2008). In the hyporheic zone (Supplementary Table 3), Mg^2+^ was significantly correlated with K^+^ and Ca^2+^, while Mg^2+^ and Ca^2+^ were significantly negatively correlated with Cl^–^ and significantly correlated with HCO_3_^–^. Previous studies indicated that HCO_3_^–^, Ca^2+^, and Mg^2+^ ions in this groundwater originated from the dissolution of calcite and dolomite (Szramek et al., 2007). In summary, a relatively strong common relationship exists between the ions in the study area, indicating that they have the same origins.

### 3.2. Analysis and comparison of microbial composition structure and diversity

#### 3.2.1. Species relative abundance and diversity in environmental communities

**FIGURE 3:**
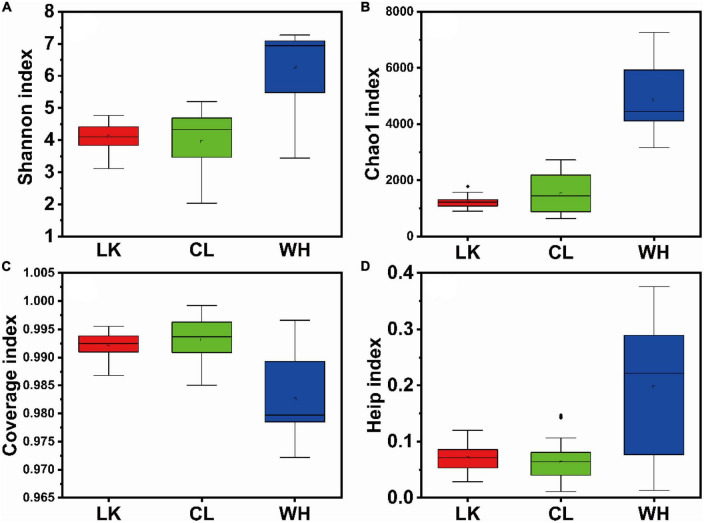
Box plots of alpha diversity indices among samples from multiple regions: **(A)** Shannon, **(B)** Chao1, **(C)** coverage, and **(D)** Heip. The horizontal axis is the division of regions, “LK” represents Longkou city, “CL” represents Cele County and “WH” represents Wuhan city, and the vertical axis shows the size and distribution of the corresponding indices for each regional sample.

A total of 26 groundwater samples were analyzed by high-throughput sequencing in Longkou, Cele, and Wuhan, and the valid sequences obtained were 1,494,357, 1,966,588, and 1,429,378, the numbers of OTUs were 3,789, 4,808, and 14,443, respectively, and these OTUs were clustered at 97% similarity. The coverage values of all samples were greater than 0.97 (Figure 3C), indicating that the sequencing depth was sufficient and the data results were reliable.

Microbial diversity can be indicated by Chao1 (Chao, 1987), ACE, Shannon, and Heip. The Chao1 (Figure 3B) and ACE indices were proportional to community richness and the Shannon index (Figure 3A) and were proportional to community diversity (Biers et al., 2009). The Heip index (Figure 3D) was proportional to community evenness (Heip and Engels, 1974). Community richness refers to the number of species in the microbial community. Community evenness refers to the distribution of the number of individuals of all species in a community or environment and is a measure of the closeness of different species in number. Community diversity is a comprehensive index reflecting species richness and evenness. Therefore, a microbial population structure is usually described with “community richness,” “community diversity,” and “community evenness” collectively WH showed the highest indices, indicating that the abundance and diversity of the microbial community structure in the hyporheic zone were the highest, and the number of bacteria was evenly distributed. The indices of the LK and CL samples were not significantly different, which indicated that the abundance and diversity of the microbial community structure in arid and coastal areas were similar, and the distribution of flora was uneven. Although the average values of the LK and CL indices were similar, the CL indices were relatively scattered, while the LK indices were relatively concentrated, indicating that the response of the community structure in the arid areas to environmental changes was more intense than that in the coastal areas. The microbial community structure in the hyporheic zone changed the most. In the coastal zone, community richness and diversity increased horizontally from DZ1 to DZ4 and vertically with increasing depth, while community richness and diversity increased from C1–C5 to C6–C10 in the arid zone and from W7 to W2 in the hyporheic zone.

#### 3.2.2. Variability in microbial composition among communities in different habitats

A comparison of microbial communities from different regions can be performed using beta diversity to quantify the similarities and differences between microbial samples. To study the similarity or differences in the relationships between different sample community structures, cluster analysis can be performed on a sample community distance matrix to construct a sample hierarchical clustering tree (White et al., 2010). The stratified clustering results showed that the groundwater samples were divided into three major categories based on their microbial composition (Supplementary Figure 1). It is divided into three regions according to the different sampling areas, where DZ1–DZ4 in the coastal zone and C1–C10 in the arid zone had more similarities than W2–W7. The similarity of the coastal zone was classified according to sampling locations, and the similarity of the arid zone was roughly classified according to a north-south trend, while the hyporheic zones W3, W4, and W5 had higher similarities.

**FIGURE 4:**
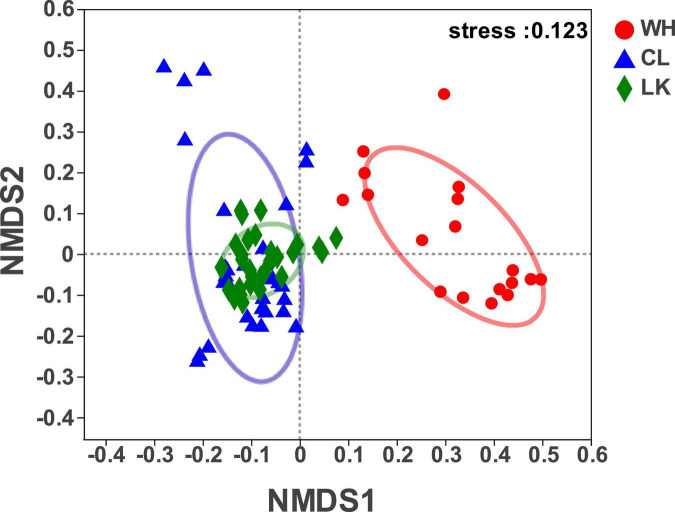
Bray – Curtis-based algorithm showing differences in community composition between samples from each study area of Longkou city (LK, green), Cele County (CL, blue) and Wuhan city (WH, red).

NMDS can reveal classifications based on the Bray – Curtis algorithm for elucidating similarities or differences in the community composition of groundwater samples and can qualitatively explain differences in microbial communities (Okubo and Sugiyama, 2009). It reflects species information contained in samples in the form of points in a multidimensional space, while the degree of difference between different samples is indicated by the distance between points, ultimately producing a spatially located point map of the samples. The results showed (Figure 4) that at the class level, when stress = 0.123, the structural distribution of microbial communities in the three regions had typical regional characteristics, and although the stress value was higher than 0.1, it still had a certain analyticity. The distribution of microorganisms in groundwater samples from the coastal and arid zones was relatively close, and many crossover areas existed, reflecting the similar composition of the microbial communities in the two regions. The microbial samples in the hyporheic zone were independently distributed on the right side of the image, indicating that the microbial community composition in the hyporheic zone was different from that of the region, suggesting that the hyporheic zone groundwater has unique microbial diversity. Thus, the microbial community structure in the coastal zone was similar to that in the arid zone, and both differed from that of the hyporheic zone.

#### 3.2.3. Comparison of the taxonomic composition of microbial communities

A total of 19,489 OTUs were identified by 16S rDNA high-throughput sequencing analysis in all groundwater samples from the three regions. The overall groundwater sample included 69 phyla, 215 classes, 550 orders, 882 families, and 1717 genera. To ensure that the relevant analyzed taxa were in a stable state in the microbial community, only OTUs that accounted for more than 2% of all samples were considered in the community composition analysis, and the rest were classified as Others. Among them (Supplementary Figure 2), *Pseudomonadota* (54.83%), *Bacteroidota* (7.96%), *Actinobacteriota* (4.17%), *Chloroflexota* (3.72%), *Cyanobacteria* (3.37%), *Bacillota* (3.21%), and *Patescibacteria* (2.54%) were ranked among the top 7 of the overall OTU table.

Further refinement of the taxonomic level of the microbial community and comparison revealed variations in the percentage of major microorganisms at each sampling site. In the coastal zone, the microbial community composition of the groundwater samples in the transitional zone between seawater and brackish water was quite different. The dominant genera of DZ10, DZ9, DZ6, and DZ3 were similar, while those of DZ4, DZ5, DZ1, and DZ2 were similar. Among them, *Lentibacter*, *Aestuaricicus*, and *Marivita* were dominant in seawater. The relative abundance of *Limnohabitans* and *Malikia* in DZ1, DZ2, DZ4, and DZ5 was relatively high, and the content of unknown bacteria in the deep aquifers was also high (DZ3 > DZ6 > DZ9). The content of hydrogenophaga in DZ2, DZ9, and DZ7 was high. In the arid zone, some genera were widely present in freshwater and brackish water; for example, the abundance of *Hydrogenophaga* in C3, C4, and C8 was very high, and the abundance of *Gallionella* in C1, C2, C7, C8, C9, and C10 was also high. Some genera had relatively high contents only in fresh water, such as *Phylotenera*, *Seminibacterium*, and *unclassified_ f__ Comamonadaceae* (Unclassified members of the *Comamonadaceae* family), *clostridium_sensu_stricto_2*. *Acinetobacter*, and *Acetobacteroides*. The genera that only had high contents in brackish water included *Sulfurimonas*, *Pseudomonas*, *Flavobacterium*, *Pseudomonas*, and *unclassified_f__Gallionellaceae* (Unclassified members of the *Gallionellaceae* family). In the hyporheic zone, the dominant genera of W3, W4, and W5 were relatively similar. The hydrogeochemistry of the subsurface flow zone made the microbial community structure more similar. At the genus level, *Limnohabitans*, *Flavobacterium*, and *Exiguobacterium* were relatively abundant in the W1 river water samples. *norank_f__norank_o__Chloroplast* had the highest content at W2, accounting for 52.41% of the total abundance. *Desulfurivibrio*, *Pseudomonas* and some other unclassified genera showed high relative abundances at W3, W4, and W5. Therefore, several microorganisms were present at almost every site, and these were more abundant than the others, but in general, the microorganisms in each study area could be categorized into a fixed number of species.

#### 3.2.4. Variation in the percentage of major microorganisms with sampling point

**FIGURE 5:**
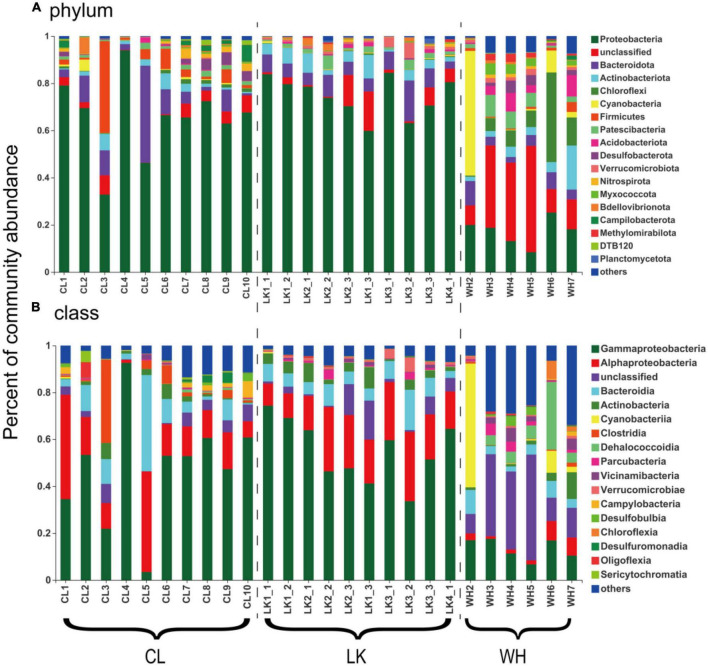
The bar graphs represent the relative abundance of dominant populations and their distribution among samples at the **(A)** phylum and **(B)** class taxonomic levels for groundwater samples from multiple areas. The horizontal axis shows the division of groundwater samples, where “CL” represents samples from Cele County. “LK” represents the sample from Longkou city, where “1_1” represents groundwater collected at the shallow level, “1_2” represents the middle level and “1_3” represents the deep level. “WH” represents the groundwater samples collected in Wuhan city. The vertical axis represents the percentage abundance of various microorganisms in the community.

Comparison at the phylum taxonomic level revealed a trend in the percentage of microorganisms based on point location (Figure 5A). Microbial community composition analysis was performed according to region. Horizontally, in the coastal zone from DZ1 to DZ4, a trend in the percentage of *Pseudomonadota* was not clear, and it consistently remained at approximately 80% in the shallow groundwater at the same depth, and the percentage of *Bacteroidota* remained at approximately 6% in all samples except DZ3-2 (17%). Longitudinally, the percentage of *Pseudomonadota* gradually decreased with increasing depth (80 –65%), and the percentage of *Bacteroidota* first increased and then decreased. A comparison from south to north in the arid zone showed that the percentage of *Pseudomonadota* did not vary much (averaging approximately 60%), while C3 had up to 40% Bacillota and C5 had up to 41% *Bacteroidota*. The percentage of *Pseudomonadota* in the hyporheic zone at W3–W5 decreased (20–10%), and most of the microorganisms were unclassified, with up to 53% *Cyanobacteria* in W2 and up to 38% *Chloroflexota* in W6. The analysis was repeated at the taxonomic class level (Figure 5B). The proportion of *Alphaproteobacteria* gradually increased horizontally from DZ1 to DZ4 (10–30%), and the proportion of *Bacteroidia* remained at approximately 7% in all samples except DZ3-2 (17%). Longitudinally, the percentage of *Gammaproteobacteria* gradually decreased with increasing depth (70–40%), and the percentage of *Bacteroidia* first increased and then decreased. A comparison from south to north in the arid zone showed a significant increase in the proportion of *Gammaproteobacteria* (30–55%), a decrease in the relative proportion of *Alphaproteobacteria*, and a high proportion of *Clostridia* in C3 (36%) and *Bacteroidia* in C5 (42%). The share of *Gammaproteobacteria* in the hyporheic zone at W3–W5 decreased (20%–10%), and most of the microorganisms were unclassified (30–40%), with up to 53% *Cyanobacteria* in W2 and up to 28% *Dehalococcoidia* in W6. Thus, both coastal and arid zones were dominated by *Pseudomonadota* as the dominant microorganisms (74.59 and 65.46%, respectively), while the abundance of major populations in the hyporheic zone was relatively moderate.

### 3.3. Correlation analysis of environmental variables with the microbial community

#### 3.3.1. Response of community richness and diversity to water quality parameters

The Pearson correlation analysis was carried out for alpha diversity indicators (Chao1, Shannon) and water chemistry variables (Supplementary Tables 4–6). In the coastal zone (Supplementary Table 4), Chao1 was strongly correlated with K^+^ (*r*^2^ = 0.804, *p* < 0.01), and the Shannon index was negatively correlated with Ca^2+^ (*r*^2^ = −0.652, *p* < 0.05), NO_3_^–^ (*r*^2^ = −0.694, *p* < 0.05), and HCO_3_^–^ (*r*^2^ = −0.633, *p* < 0.05). It can be seen from the analysis of the physical and chemical properties of the site that K^+^ is the least of the main cations, while Ca^2+^ is the most. Therefore, as a necessary nutrient for microorganisms, K^+^ shows a high positive correlation with the changing trend in microbial number. However, nitrate pollution and bicarbonate pollution in coastal areas will reduce the diversity of microbial communities. In the arid zone (Supplementary Table 5), pH showed a strong correlation with the Chao1 (*r*^2^ = 0.771, *p* < 0.01) and Shannon indices (*r*^2^ = 0.808, *p* < 0.01). Ca^2+^ showed a negative correlation with the Chao1 (*r*^2^ = −0.633, *p* < 0.05) and Shannon indices (*r*^2^ = −0.923, *p* < 0.01). Moreover, ORP showed a negative correlation with the Chao1 index (*r*^2^ = −0.639, *p* < 0.05), and NO_3_^–^ showed a negative correlation with the Shannon index (*r*^2^ = −0.823, p < 0.01). Groundwater in Cele arid area is neutral to weak alkaline. With the increase in pH, the number and diversity of microbial communities increase. In the hyporheic zone (Supplementary Table 6), EC (*r*^2^ = 0.966, *p* < 0.01), TDS (*r*^2^ = 0.966, *p* < 0.01), Ca^2+^ (*r*^2^ = 0.947, *p* < 0.01), and Mg^2+^ (*r*^2^ = 0.941, *p* < 0.01) showed a significant correlation with the Shannon index, and DO show a negative correlation with the Shannon index (*r*^2^ = −0.826, *p* < 0.05). With the increase in salinity and electrical conductivity of groundwater, the diversity of microbial communities increased. However, DO will limit the number and diversity of microbial communities, which is the same as previous studies (Chen, 2020). Of course, the problems of sparse samples and small sample size will cause a certain deviation in the results of correlation analysis (Campbell and Kirchman, 2013). In general, environmental factors affect the abundance and diversity of groundwater communities in different ways.

#### 3.3.2. Responses between sampling locations and environmental factors

**FIGURE 6:**
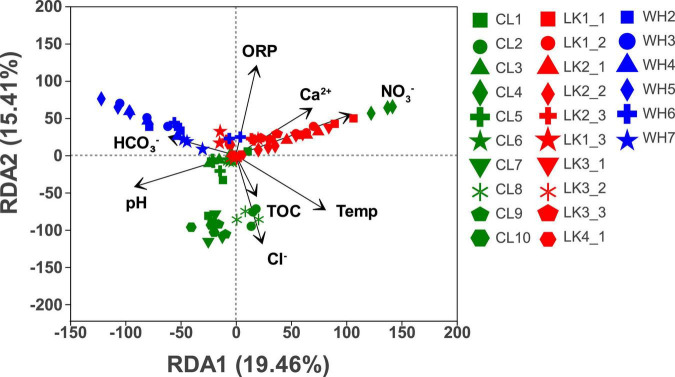
Redundancy analysis of sample community composition and environmental factors in each study area: Longkou City (red), Cele County (green) and Wuhan City (blue). The values on the horizontal and vertical axes are the percentages explained by the two largest corresponding axes. Symbols represent samples, and arrows represent environmental factors.

To reduce the number of environmental variables in the redundancy analysis, variables with high covariance were iteratively screened and removed using VIF variance inflation factor analysis (Stine, 1995). When the initial environment variables exhibit strong covariance with large VIF values (above a threshold of 10), they are chosen to be removed. As a result, a total of six variables causing strong covariance (EC, TDS, K^+^, Na^+^, Mg^2+^, and SO_4_^2–^) were removed, and eight variables (temperature, pH, ORP, TOC, Ca^2+^, Cl^–^, NO_3_^–^, and HCO_3_^–^) were retained. After the environmental factors at the three sites were screened by VIF variance-inflated factor analysis, RDA was performed to investigate whether and to what extent the environmental factors affected the community structure of microorganisms. As shown in Figure 6, the first axis explains 19.46%, while the second axis explains 15.41%. HCO_3_^–^, Cl^–^, and NO_3_^–^ were more significant in affecting the bacterial community structure.

The groundwater physicochemical parameters show three main gradients. In the coastal zone, with increasing depth at the sampling points and with gradual decreases in the distance from the coast, the influence of three environmental factors (Ca^2+^, ORP, and NO_3_^–^) on groundwater gradually decreased. In the arid zone, the response to four environmental factors (TOC, Cl^–^, temperature, and pH) was more pronounced at sites located in the north than in the south. In the hyporheic zone, points W3–W5, which were located in the middle of the sampling area, gradually showed an increase in the influence of HCO_3_^–^ as the distance from the riverbank increased. Therefore, the dominant environmental factors varied from place to place, and there was a clear trend in the changes.

#### 3.3.3. Major environmental factors and microbial responses to them in different aquifers

The dominant environmental factors in the coastal zone are shown in Supplementary Figure 3. The microbial communities in DZ1-1 as well as DZ1-2 were not affected by seawater intrusion and were more influenced by temperature and NO_3_^–^, specifically *Limnohabitans*, which had a significant correlation with NO_3_^–^. The microbial communities in the deep aquifer were mainly influenced by temperature, HCO_3_^–^, and NO_3_^–^, specifically *Hydrogenophaga*, *Azospirillum*, and HCO_3_^–^ had a significant correlation. *Lentibacter* and *Aestuariicoccus* showed significant negative correlations with temperature and HCO_3_^–^ and NO_3_^–^ levels; however, they showed significant positive correlations with other ions concentrations. The dominant environmental factors in the arid zone were pH, TOC, Ca^2+^, and NO_3_^–^ (Supplementary Figure 4). Among them, the microbial community structures of the freshwater sites (C1 and C2) and brackish water sites (C7, C8, C9, and C10) were more influenced by pH; specifically, *Gallionella* was significantly correlated with pH. C4 was strongly influenced by TOC, Ca^2+^, and NO_3_^–^, specifically *Hydrogenophaga*, *Methylotenera* showed a significant correlation with TOC, Ca^2+^, and NO_3_^–^. The C5 and C6 sample sites were more affected by Ca^2+^ and NO_3_^–^, specifically *Pseudorhodobacter*, and *Flavobacterium* showed a significant correlation with Ca^2+^ and NO_3_^–^. The dominant environmental factors in the hyporheic zone were EC, DO, pH, ORP, temperature, and TOC (Supplementary Figure 5). Among them, the microbial community structure of sample sites W3, W4, and W5 in the river manholes was more influenced by EC and ORP; specifically, the unclassified bacteria *Desulfurivibrio* and EC and ORP had significant correlations. The microbial community structures of the river W1 and W2 sample sites, which were closer to the river, were more influenced by DO and pH; specifically, *Chloroplast* and *Flavobacterium* were significantly correlated with DO and pH. The groundwater sample sites W6 and W7 were far from the river and were more influenced by TOC, temperature, and EC; specifically, many unclassified bacterial families were significantly correlated with TOC, temperature, and EC. The response of specific microorganisms to environmental factors reveals that the effect of these factors on the microorganisms were distinct from place to place.

### 3.4. Microorganisms that differed significantly in relative abundance among groups

**FIGURE 7:**
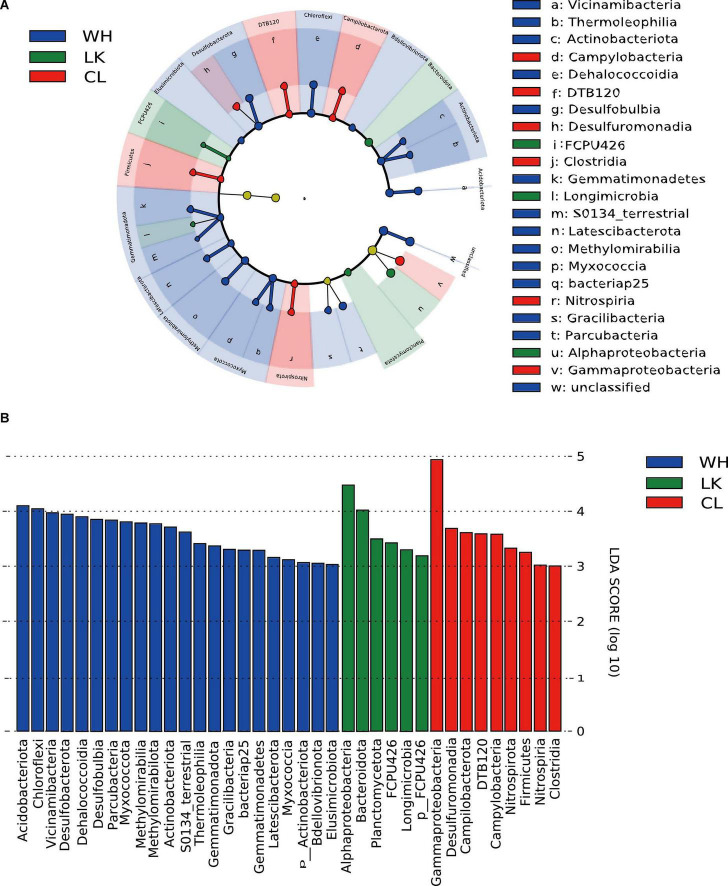
**(A)** Branching diagrams representing the phylogenetic distribution of groundwater microorganisms at the class taxonomic level in the three different study areas. Biomarkers of significantly different species are colored based on the different groups, and the species names corresponding to the biomarkers are displayed on the right side of the figure, with the letter numbers corresponding to panel **(B)** significantly different species with LDA scores greater than a preset value of 3. The colors of the bars represent the respective groups, and the lengths represent the LDA score.

To determine which microorganisms differed across multiple subgroups in different regions, we performed a LEfSe, looking for biomarkers that were significantly different from group to group. The LDA plots (Figure 7) show the magnitude of significant differences, with larger differences being more significant. The LDA significance threshold was chosen to be 3.0 for the calculation. This branching diagram (Figure 6) represents the significant microbial differences between LK (green), CL (red), and WH (blue) at the phylum and class taxonomic levels for the three sites.

The differential microorganisms at the phylum classification level were as follows: in the coastal zone, *Bacteroidota* (7.53%), *Planctomycetota* (0.40%); in the arid zone, *Fimicutes* (6.60%), *Campilobacterota* (1.50%), *Nitrospirota* (1.41%), and *DTB120* (0.59%); and in the hyporheic zone, *Desulfobacterota* (2.23%), *Chloroflexota* (11.73%), *Acidobacterota* (4.20%), *Myxococcota* (2.49%), *Methylomirabilota* (1.56%), *Gemmatimondota* (0.94%), *Bdellovlbrionota* (0.56%), and *Actinobacteriota* (5.95%). The differential microorganisms at the class taxonomic level were *Alphaproteobacteria* (19.30%) in the coastal zone; *Clostridia* (5.38%), *Nitrospiria* (0.99%), *Campylobacteria* (1.50%), and *Desulfuromonadia* (0.95%) in the arid zone; and *Dehalococcoidia* (8.05%), *Vicinamibacteria* (2.65%), *Thermoleophilia* (1.20%), *Desulfobulbia* (1.38%), *Gracilibacteria* (1.36%), *Methylomirabilia* (1.56%), *Parcubacteria* (2.22%), and *Myxococcia* (0.52%) in the hyporheic zone. As the OTUs were clustered at 97% similarity, the species level was not very accurate in high-throughput sequencing, but it can be used as a reference. Further subdivision into species taxonomic levels revealed that the main differential microorganisms in the coastal zone were *Proteobacteria*, *Comamonadaceae*, *Alphaproteobacteria*, *Hydrogenophaga*, and *Limnohabitans*. The main differential microorganisms in the arid zone were *Gallionella*, *Rhodobacteraceae*, *Bacillota*, and *Sideroxydans*. The main differential microorganisms in the hyporheic zone were *Chloroflexota*, *Myxococcota*, *Desulfobacterota*, and *Vicinamibacterales*. Microorganism analysis by the level of classification in each location revealed that there were clear differences in the dominant microorganisms.

### 3.5. Comparative analysis of interactions within microorganisms

**FIGURE 8:**
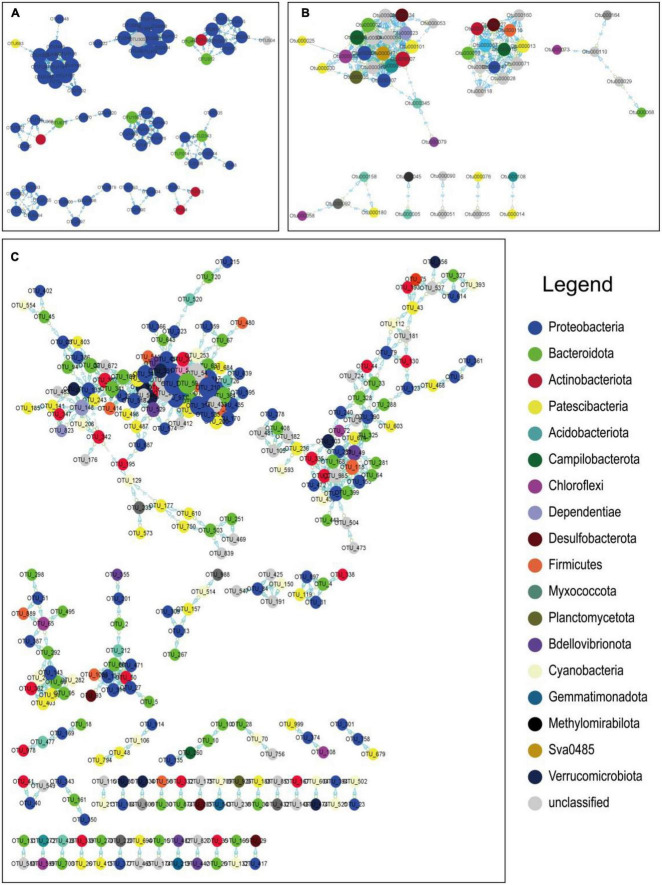
Molecular ecological network of the study area based on OTUs in panel **(A)** Longkou city, **(B)** Cele County, and **(C)** Wuhan city. The nodes in different colors represent different species of microorganisms at the phylum classification level, with positive correlations between nodes indicated as “pp” and negative correlations as “np”, and the connectivity of nodes is indicated by the relative size of the circles in the diagram. The connectivity of the nodes is indicated by the relative size of the circles in the diagram. Some of the main nodes representing microbial species are shown on the right.

MENA (Deng et al., 2012) is used to depict the overall structure of a microbial community and to identify key microorganisms (Figure 8). In an ecological network, each element (organism or gene) can be described as a node in the network, and the relationship between them can be described as an edge in the network. Molecular ecological networks were constructed for the three different geographical environments based on microbial communities at the phylum taxonomic level. In the networks, the number of coastal zone nodes is 71, the number of links is 192, the number of positive correlations is 192, and the number of negative correlations is 0. For the arid zone, there are 51 nodes, 198 links, 197 positive correlations, and 1 negative correlation. The number of nodes in the hyporheic zone is 279, the number of links is 694, the number of positive correlations is 694, and the number of negative correlations is 0. It is generally accepted that the greater the number of edges a node is connected to, the more pivotal its position in the network.

In the coastal zone, the main OTUs belonged to Proteobacteria. According to the taxonomic table breakdown, the taxonomic level of the class was mainly *Gammaproteobacteria* and *Alphaproteobacteria*, the taxonomic level of the order was mainly *Burkholderiales*, and the taxonomic level of the family was mainly *Rhodocyclaceae* and *Comamonadaceae*. In the arid zone, the main OTUs belonged to *Actinobacteriota*, *Proteobacteria*, *Campilobacterota*, *Bacteroidota*, *Patescibacteria*, and *Desulfobacterota*. In the hyporheic zone, the main OTUs belonged to *Proteobacteria*, *Cyanobacteria*, and *Bacteroidota* and were subdivided according to the taxonomic table. The taxonomic level of the class was mainly *Gammaproteobacteria* and *Cyanobacteria* and *Bacteroidia*, and the taxonomic level of the order was mainly *Chloroplast*. The microorganisms corresponding to the major OTUs represent their dominant position and function in the community, and therefore, the biogeochemical processes embodied in each location differ as a result.

## 4. Discussion

### 4.1. Spatial distribution affects physical and chemical indicators

Geographical location has a significant effect on the composition of ions and their corresponding distributions (Zhou B. et al., 2021; Meharg et al., 2022).

The Longkou study area was located in a hydrogeological area containing low mountains and hills in eastern Shandong, a coastal plains subarea, and an intermountain accumulation plains area. According to groundwater origin, it can be divided into three types: carbonate rock fissure karst water, bedrock fissure water, and loose rock pore water. The study area was strongly affected by seawater intrusion. Therefore, the hydrochemical type of the groundwater was mainly the HCO_3_. Cl-Ca and HCO3. Cl-Na. Ca type, the TDS was generally less than 1,000 mg/L, and the TDS of the groundwater in the seawater intrusion areas was relatively high, generally between 2,100 and 5,900 mg/L.

The study area in the Cele arid area showed groundwater characteristics that are typical of a dry alluvial fan area, and the groundwater flow direction and surface runoff flow direction were from south to north. The TDS of the groundwater north of Cele arid area was higher than that in the south. The cation types were mainly Na^+^ and Ca^2+^. The anion types were mainly Cl^–^ and SO_4_^2–^. The concentration of the main ions increased gradually with the direction of groundwater runoff from south to north. The concentrations of Cl^–^, SO_4_^2–^, and HCO_3_^–^ anions increased gradually in the direction of groundwater runoff.

The Wuhan study area was located at an accumulation bank of the Yangtze River and belonged to an accumulation plains area in the middle reaches of the Yangtze River. The terrain was flat, open, and slightly inclined from north to south. The subsurface flow zones usually exhibited complex characteristics that were influenced by various physical, chemical and biological processes. The overall TDS of the study area was low, and the HCO_3_^–^ level was high, but the chemical indicators did not show significant changes with increasing distance from the riverbank, and the pH decreased slightly.

According to the comparison shown in Figure 2, the variation in anion concentration was more severely influenced by the environment (Yang et al., 2020; Yuan Y. et al., 2020; Xu et al., 2022). Cl^–^ concentration was high in both the coastal and arid zones due to the presence of seawater intrusion in the coastal zone and evaporation in the arid zone (Idowu et al., 2022; Yin et al., 2022). The concentration and percentage of HCO_3_^–^ were high in the hyporheic zone because of the presence of strong biogeochemical reactions that led to a large depletion of other ions (Korrai et al., 2021; Xu et al., 2021). NO_3_^–^ concentrations were abnormally high in the coastal areas because near-shore estuaries are environmentally sensitive zones that are prone to nutrient deposition, and seawater intrusion leads to excessive nitrate loads in the groundwater (Han et al., 2015; Cao Y. et al., 2020; Kaown et al., 2021).

In general, the three study areas represented different environments and thus exhibited different water chemistry characteristics. In the coastal zone, due to seawater intrusion, water type transformation occurred; in the arid zone, under arid conditions, strong evaporation led to an increase in ion concentration and promoted mineralization; in the hyporheic zone, strong interactions and the weathering of salt rocks combined to promote ion concentration changes.

### 4.2. Physicochemical indicators affect microbial community structure

Physicochemical parameters become potential influencing factors for microbial communities (Albers et al., 2015; Salazar et al., 2020; Nayak et al., 2021; Meharg et al., 2022; Zhang et al., 2022).

In the Wuhan subsurface flow zone, the groups that had relatively high abundance at the phylum level were *Proteobacteria*, *Cyanobacteria*, *Chloroflexota*, *Bacteroidota*, and *Actinobaciota*. The environmental factors that had strong impacts on the microbial community structure were EC, DO, pH, ORP, T, and TOC. In particular, the microbial community structure of the W3, W4, and W5 sampling points in the floodplain was greatly affected by EC and ORP. The microbial community structure of the W1 and W2 sample sites near the river water was greatly affected by DO and pH. The groundwater sampling points W6 and W7, which are far from the river, were greatly affected by TOC and T.

The microbial diversity of the brackish water group in the northern Cele arid area was higher than that of the freshwater group. At the phylum level, *Proteobacteria*, *Bacteroidota*, and *Bacillota* had higher abundances. At the family level, *Gallionellaceae* and *Comamondaceae* had higher abundances. CCA between microbial community structure and environmental factors at the genus level showed that pH, TOC, Ca^2+^, and NO_3_^–^ had significant effects on microbial community structure. Among them, the microbial community structure of the fresh water sites (CL1 and CL2) and brackish water sites (CL7, CL8, CL9, and CL10) was greatly affected by pH, while that of the fresh water site CL4 was greatly affected by TOC, Ca^2+^, and NO_3_^–^. The samples from CL5 and CL6 at the saline water sites were greatly affected by Ca^2+^ and NO_3_^–^.

The results of microbial alpha diversity analysis in the coastal area of Longkou showed that the richness index of the seawater was lower than that of groundwater in the brackish freshwater transition zone, and the salinity of seawater was high, which could inhibit the growth of many bacteria. Beta diversity analysis verified that seawater intrusion significantly affected the microbial community structure and had the greatest impact on the middle aquifer. The microbial community structure of groundwater in the transitional zone between seawater and brackish water was quite different. *Pseudomonadota* had an absolute advantage at the phylum level. At the order level, *Rhodobacterales* was dominant in the seawater, and *Burkholderiales* dominated the groundwater.

Based on the analysis in the results section, the coastal areas clearly showed different microbial community structures than the arid areas, although they were at similar salinity values. Microbial diversity was most abundant in the subsurface flow zone of the study area, that of the arid area was similar to the coastal area, and the microbial population in the arid area was more unstable due to changes in habitat. The arid area had relatively high Cl^–^ and SO_4_^2–^ concentrations, and in contrast with the other study areas, its TOC levels exceeded the TOC limit due to evaporation. The main microorganisms at this site were the families *Gallionellaceae* and *Comamonadaceae* of the *Burkholderiales* order. The coastal area had relatively high NO_3_^–^ and Cl^–^ concentrations due to seawater intrusion, but the main microorganisms in the area were the *Comamonadaceae* family and *Rhodocyclaceae* family of *Burkholderiales* order. In contrast, the hyporheic zone was a low salinity area; among the ions, only HCO_3_^–^ was detected at very high concentrations (Liao et al., 2019), and the main microorganisms in this area were *Comamonadaceae* and *Desulfurivibrionaceae*.

Therefore, each region had marker microorganisms due to the specific physicochemical parameters of the area. In combination with Figure 6, it can be concluded that high concentrations of nitrate were present in the coastal zone and that the main microbial metabolism was related to denitrification, driven by the dominant microorganisms belonging to *Rhodocyclaceae* (Oren, 2014; Magri et al., 2020). High concentrations of ions were present in the arid zone, and the dominant microorganisms, *Gallionellaceae*, were associated with oxidation processes (Kadnikov et al., 2016; Yu et al., 2020). High concentrations of carbonate and very low concentrations of other ions were present in the hyporheic zone, and the main microorganisms involved in the sulfur conversion process belonged to *Desulfurivibrionaceae* (Sorokin et al., 2008; Yi et al., 2020). Thus, physicochemical parameters direct the structure of groundwater microbial communities.

## 5. Conclusion

In this study, groundwater was collected from different geographical environments. Multivariate analyses of the microbial and hydrochemical variables were carried out to provide a basis for deeper analyses of the main biochemical tendencies of different geographical environments. The results showed that microorganisms belonging to *Comamonadaceae*, which dominated at locations far from the coast, gradually decreased under the influence of salinity in the coastal zone due to the influence of seawater intrusion, and the genus *Hydrogenophaga*, which mainly carries out denitrification, corresponded to NO_3_^–^ levels, one of the main local environmental impact factors. In the arid zone, ion concentrations were elevated due to synergistic effects between long-distance groundwater transport and strong evaporation in the external environment, and a relatively large proportion of *Gallionella* was present, indicating that iron redox processes occurred in the local groundwater. The hyporheic zone had intense biogeochemistry, with high ORP variations and fairly high HCO_3_^–^ concentrations and a wide variety and number of microorganisms, mainly *Desulfurivibrio*, which promotes sulfur conversion, and several species of bacteria that are photosynthetically active.

The data from this study and subsequent analyses reveal significant differences in the hydrochemical variables and microbial structures of the different geographical environments and indicate a clear biochemical tendency for microbial–hydrochemical variable interactions in the different aquifers, which direct the formation of the groundwater environment.

## Data availability statement

The data presented in this study are deposited in the NCBI repository (https://www.ncbi.nlm.nih.gov/bioproject/PRJNA929086), accession number: PRJNA929086, further inquiries can be directed to the corresponding author.

## Author contributions

HD: conceptualization, methodology, and software. YZ: data curation and analysis, and manuscript writing and revision. WF: data curation and writing—original draft preparation. JL: data acquisition and modification. JZ: visualization, investigation, and supervision. CZ: software and validation. JH: writing—reviewing and editing. All authors contributed to the article and approved the submitted version.
