# Supplementary material for: Microbial community structural response to variations in physicochemical features of different aquifers
Source: Front Microbiol. 2023 Feb 9;14:1025964. doi: 10.3389/fmicb.2023.1025964 (PMC9971630; doi:10.3389/fmicb.2023.1025964)
Supplement: Supplementary file 1 [file Data_Sheet_1.docx]

Supplementary materials to

**Microbial community structural response to variations in the chemical–physical water characteristics of different aquifers**

Heng Dai^1,2^, Yiyu Zhang^1,2^, Wen Fang^1,2^, Juan Liu^1,2^, Chaowang Zou^3^, Jun Hong^4^, Jin Zhang^5, 6^*

^1^State Key Laboratory of Biogeology and Environmental Geology, China University of Geosciences, Wuhan 430078, China

^2^Hubei Key Laboratory of Yangtze Catchment Environmental Aquatic Science, School of Environmental Studies, China University of Geosciences, Wuhan 430078, China

^3^Hubei Institute of Water Resources Survey and Design, 290 South Luoshi Road, Hongshan District, Wuhan 430070, China

^4^School of Environmental Studies, China University of Geosciences, Wuhan 430078, China

^5^Yangtze Institute for Conservation and Development, State Key Laboratory of Hydrology-Water Resources and Hydraulic Engineering, Hohai University, 210098 Nanjing, China

^6^Xinjiang Institute of Ecology and Geography, Chinese Academy of Sciences, 830011 Urumqi, China

*Corresponding author: jin.zhang@hhu.edu.cn


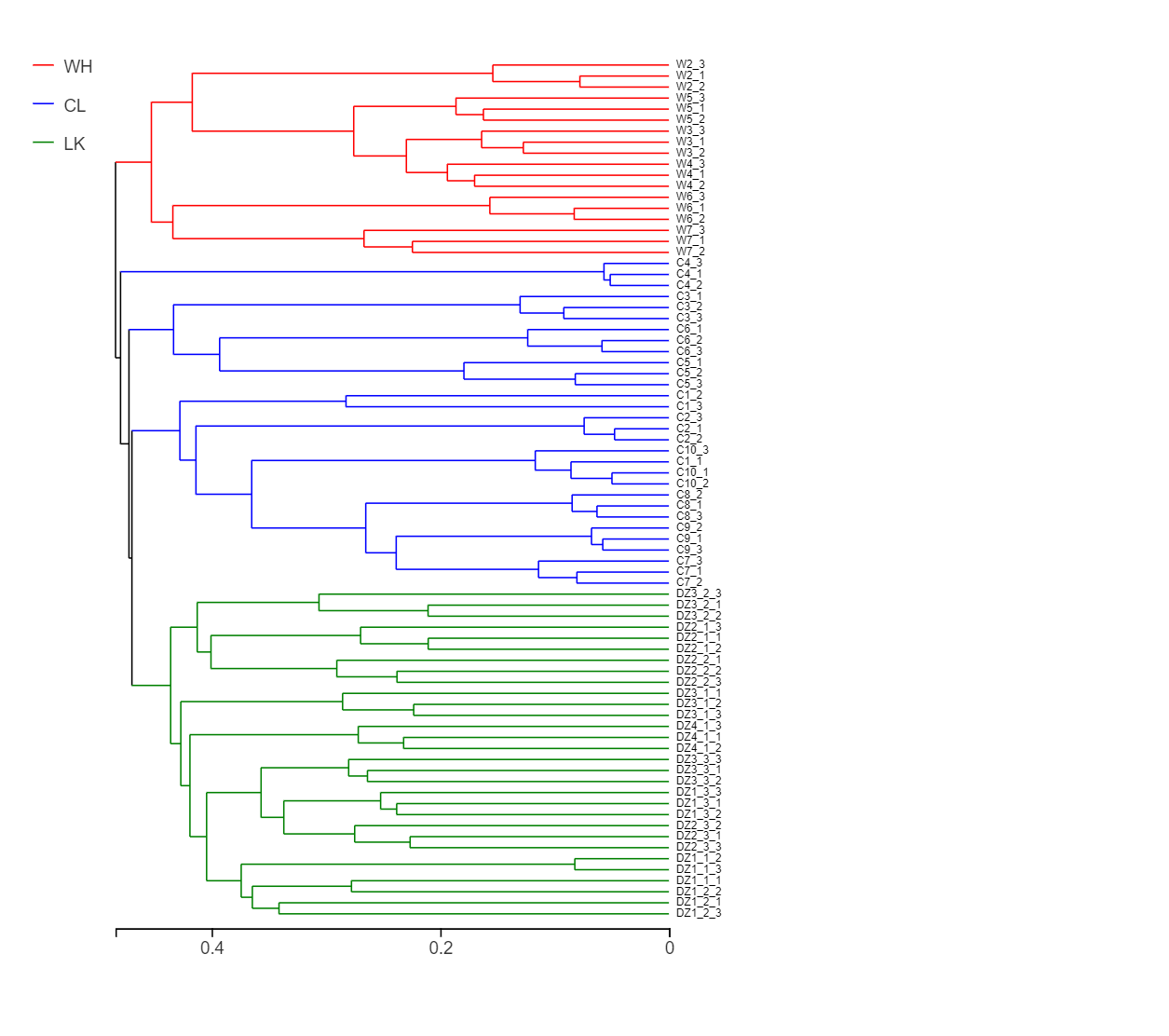


Figure S1. Hierarchical clustering of different aquifer samples by OTU classification level


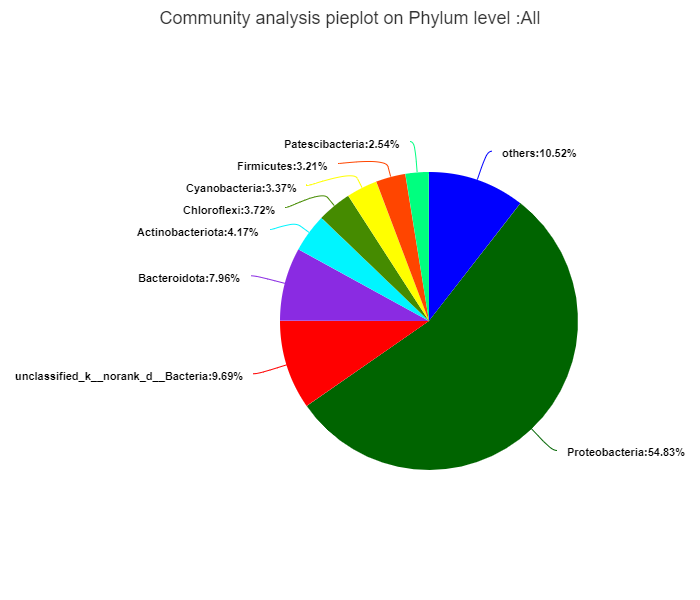


Figure S2. The pie chart represents the relative abundance of dominant bacterial communities in groundwater samples, classified at the phylum level.


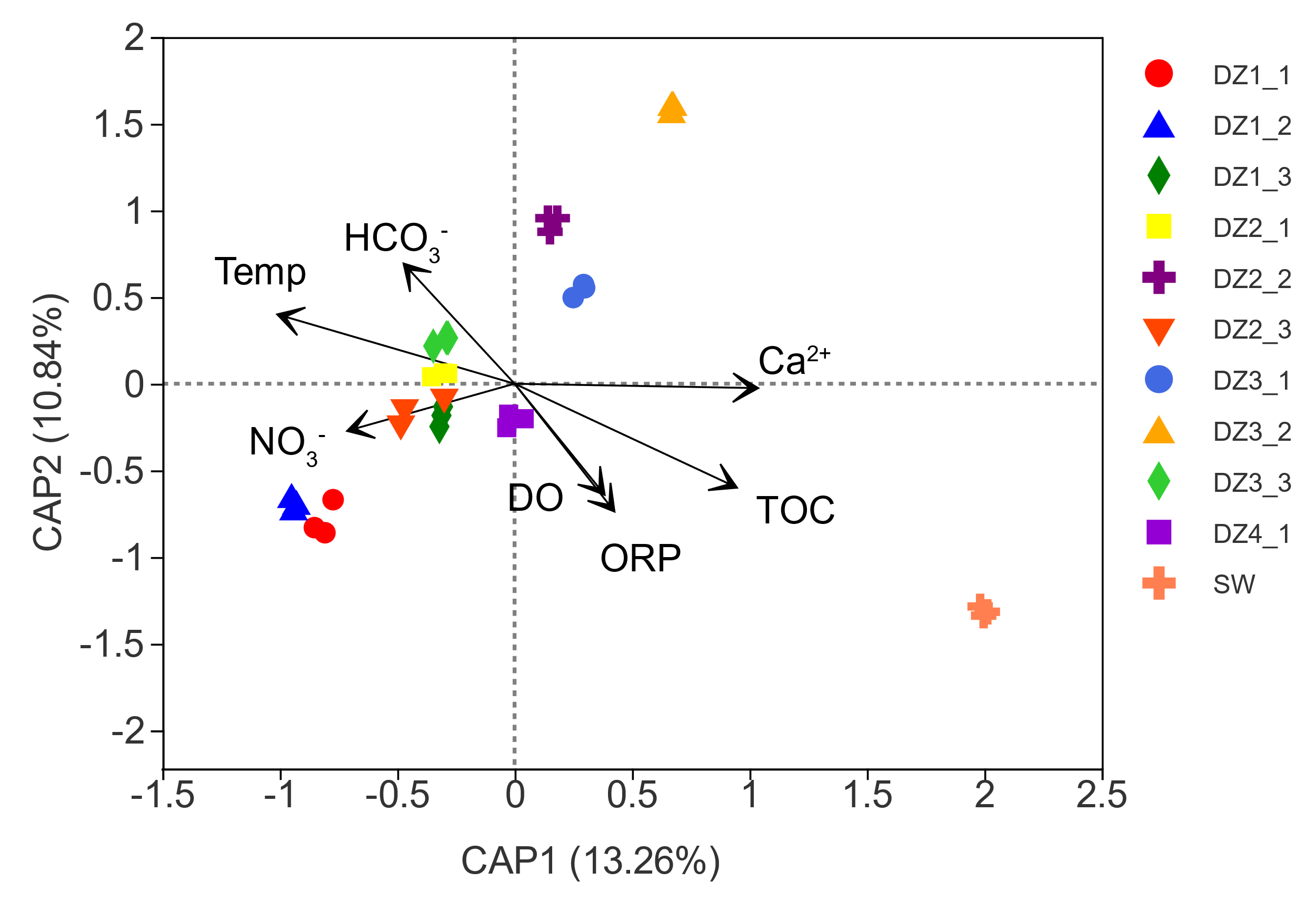


Figure S3. Distance-based redundancy analysis of the effect of environmental factors on microbial communities at the genus level in Longkou.


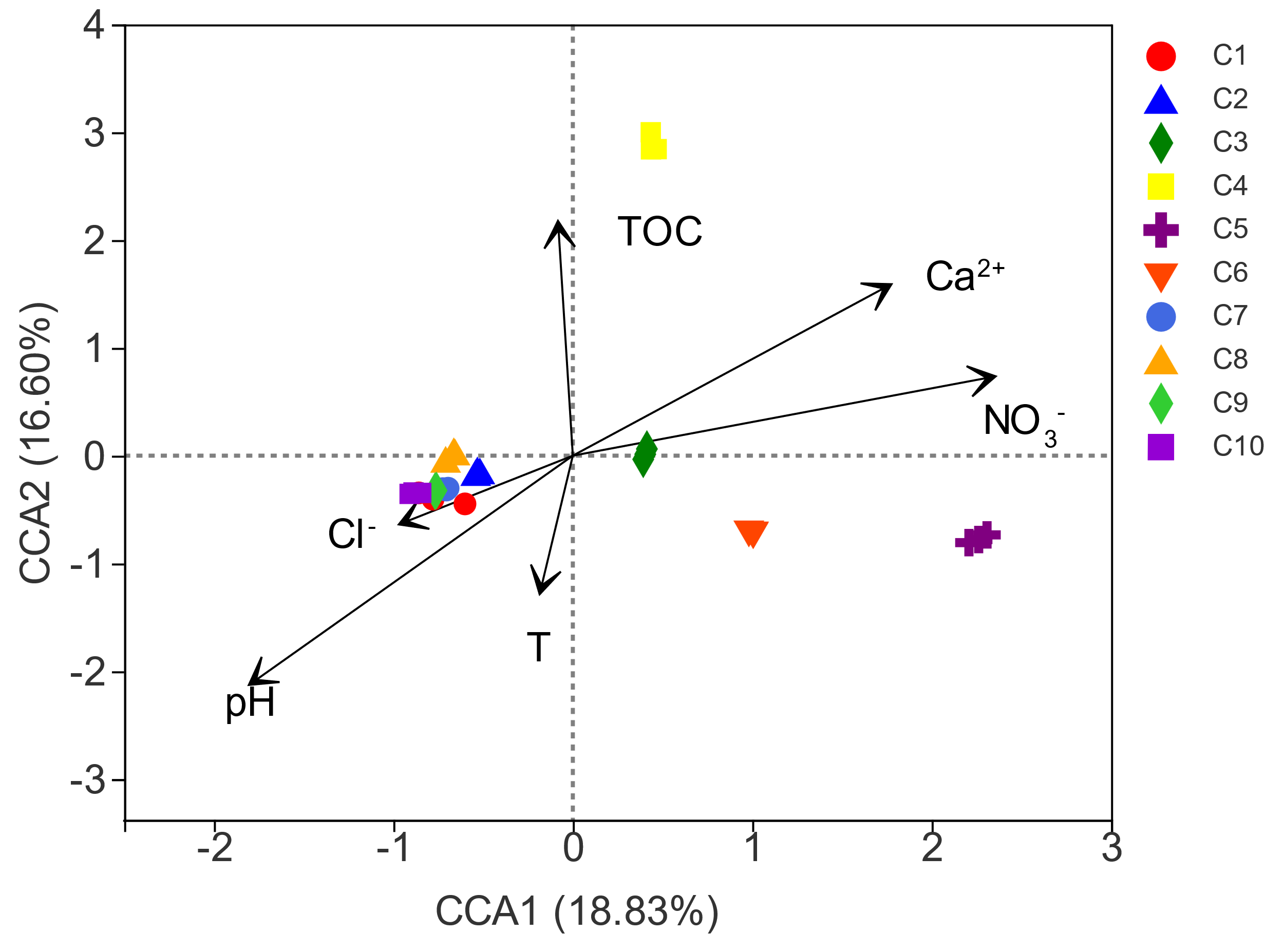


Figure S4. Canonical correlation analysis of the effect of environmental factors on microbial communities at the genus level in Cele.


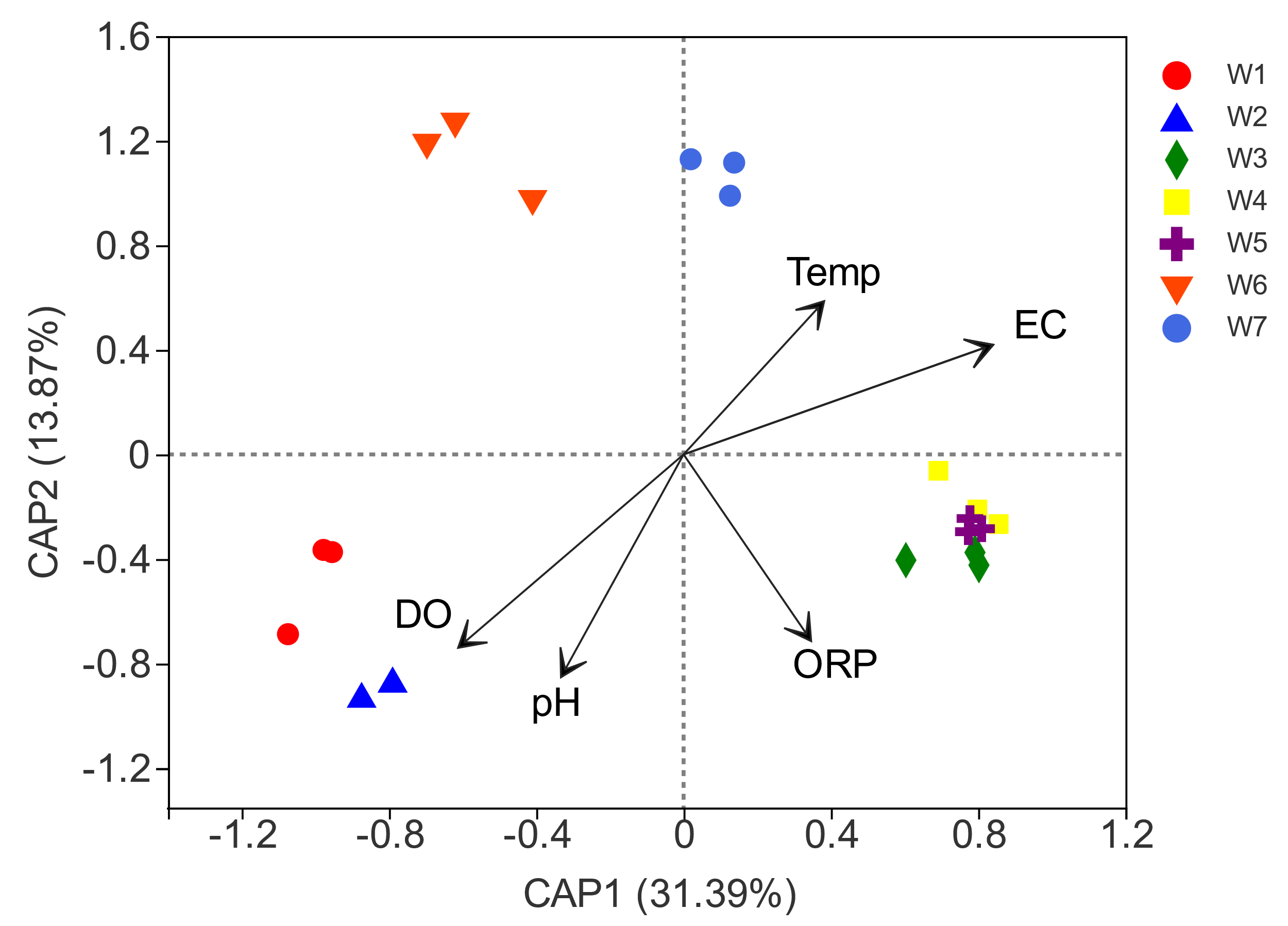


Figure S5. Distance-based redundancy analysis of the effect of environmental factors on microbial communities at the genus level in Wuhan.

**Table S1**

Pearson correlation analysis between the main physicochemical properties of water samples from Longkou

| Physical and chemical parameters | pH | DO | ORP | EC | TDS | TOC | K^+^ | Ca^2+^ | Na^+^ | Mg^2+^ | Cl^-^ | NO_3_^-^ | SO_4_^2-^ | HCO_3_^-^ |
| --- | --- | --- | --- | --- | --- | --- | --- | --- | --- | --- | --- | --- | --- | --- |
| Temp | -.426 | -.410 | .215 | .141 | .125 | .244 | .377 | .160 | .196 | .121 | -.032 | .009 | .425 | .267 |
| pH |  | .442 | -.163 | **-.671*** | **-.780**** | .050 | **-.800**** | **-.760*** | **-.759*** | -.558 | -.612 | .148 | **-.906**** | **-.755*** |
| DO |  |  | **-.706*** | -.481 | -.054 | -.214 | -.344 | .003 | .032 | -.137 | .002 | **.645*** | -.346 | .124 |
| ORP |  |  |  | .402 | -.261 | .001 | .261 | -.223 | -.450 | -.128 | -.429 | -.177 | .120 | -.327 |
| EC |  |  |  |  | .588 | -.215 | .416 | .591 | .240 | **.671*** | .372 | -.005 | **.716*** | .245 |
| TDS |  |  |  |  |  | -.312 | .383 | **.979**** | **.845**** | **.740*** | **.941**** | .151 | **.651*** | **.786**** |
| TOC |  |  |  |  |  |  | -.055 | -.334 | .005 | -.309 | -.268 | -.219 | -.113 | -.037 |
| K^+^ |  |  |  |  |  |  |  | .361 | .454 | .370 | .193 | -.234 | .**861**** | .466 |
| Ca^2+^ |  |  |  |  |  |  |  |  | **.820**** | **.664*** | **.887**** | .276 | **.643*** | **.805**** |
| Na^+^ |  |  |  |  |  |  |  |  |  | .403 | **.843**** | -.031 | .603 | **.933**** |
| Mg^2+^ |  |  |  |  |  |  |  |  |  |  | **.652*** | .119 | .608 | .286 |
| Cl^-^ |  |  |  |  |  |  |  |  |  |  |  | .027 | .433 | **.703*** |
| NO_3_^-^ |  |  |  |  |  |  |  |  |  |  |  |  | -.138 | .194 |
| SO_4_^2-^ |  |  |  |  |  |  |  |  |  |  |  |  |  | .586 |

TOC: total organic carbon; EC: electrical conductivity; TDS: total dissolved solids; ORP: Oxidation-Reduction Potential; DO: dissolved oxygen.

⁎ Indicate significant differences at *p* < 0.05. ⁎⁎ Indicate significant differences at *p* < 0.01.

**Table S2**

Pearson correlation analysis between the main physicochemical properties of water samples from Cele

| Physical and chemical parameters | pH | ORP | EC | TDS | TOC | K^+^ | Ca^2+^ | Na^+^ | Mg^2+^ | Cl^-^ | NO_3_^-^ | SO_4_^2-^ | HCO_3_^-^ |
| --- | --- | --- | --- | --- | --- | --- | --- | --- | --- | --- | --- | --- | --- |
| Temp | .354 | -.617 | .239 | .125 | -.276 | .140 | -.576 | .128 | .037 | .155 | -.555 | .125 | -.250 |
| pH |  | **-.723*** | .553 | .544 | -.304 | .485 | **-.722*** | .601 | .406 | .531 | -.616 | .519 | -.069 |
| ORP |  |  | **-.703*** | **-.638*** | -.135 | **-.645*** | .557 | **-.657*** | -.484 | -.627 | .476 | **-.648*** | -.268 |
| EC |  |  |  | **.957**** | -.001 | **.918**** | -.275 | **.953**** | **.946**** | **.972**** | -.301 | **.975**** | .532 |
| TDS |  |  |  |  | .034 | **.971**** | -.256 | **.995**** | **.950**** | **.993**** | -.183 | **.994**** | .590 |
| TOC |  |  |  |  |  | .055 | .388 | .028 | -.021 | -.018 | .356 | .077 | .520 |
| K^+^ |  |  |  |  |  |  | -.180 | **.946**** | **.926**** | **.951**** | -.017 | **.958**** | **.697*** |
| Ca^2+^ |  |  |  |  |  |  |  | -.319 | -.156 | -.260 | **.792**** | -.209 | .442 |
| Na^+^ |  |  |  |  |  |  |  |  | **.933**** | **.988**** | -.259 | **.988**** | .529 |
| Mg^2+^ |  |  |  |  |  |  |  |  |  | **.956**** | -.106 | **.953**** | .609 |
| Cl^-^ |  |  |  |  |  |  |  |  |  |  | -.244 | **.993**** | .536 |
| NO_3_^-^ |  |  |  |  |  |  |  |  |  |  |  | -.191 | .603 |
| SO_4_^2-^ |  |  |  |  |  |  |  |  |  |  |  |  | .609 |

TOC: total organic carbon; EC: electrical conductivity; TDS: total dissolved solids; ORP: Oxidation-Reduction Potential.

⁎ Indicate significant differences at *p* < 0.05. ⁎⁎ Indicate significant differences at *p* < 0.01.

**Table S3**

Pearson correlation analysis between the main physicochemical properties of water samples from Wuhan

| Physical and chemical parameters | pH | DO | ORP | EC | TDS | TOC | K^+^ | Ca^2+^ | Na^+^ | Mg^2+^ | Cl^-^ | NO_3_^-^ | SO_4_^2-^ | HCO_3_^-^ |
| --- | --- | --- | --- | --- | --- | --- | --- | --- | --- | --- | --- | --- | --- | --- |
| Temp | -.734 | **-.850*** | -.260 | .715 | .713 | .078 | .499 | .784 | -.288 | **.820*** | **-.888*** | .450 | -.467 | .692 |
| pH |  | **.833*** | .615 | -.486 | -.486 | -.405 | -.001 | -.541 | .356 | -.515 | **.823*** | -.649 | .219 | -.536 |
| DO |  |  | .446 | **-.844*** | **-.843*** | -.539 | -.533 | **-.887*** | .475 | **-.894*** | **.989**** | -.530 | .512 | **-.830*** |
| ORP |  |  |  | .054 | .055 | -.677 | .120 | -.075 | -.064 | -.195 | .468 | -.542 | .546 | -.336 |
| EC |  |  |  |  | **1.000**** | .360 | .726 | **.982**** | -.655 | **.922**** | -.802 | .147 | -.305 | **.827*** |
| TDS |  |  |  |  |  | .360 | .725 | **.982**** | -.656 | **.921**** | -.801 | .145 | -.303 | **.827*** |
| TOC |  |  |  |  |  |  | .210 | .411 | -.414 | .429 | -.475 | .193 | -.435 | .614 |
| K^+^ |  |  |  |  |  |  |  | .722 | -.096 | **.822*** | -.566 | .145 | -.694 | .574 |
| Ca^2+^ |  |  |  |  |  |  |  |  | -.655 | **.964**** | **-.853*** | .150 | -.400 | **.908*** |
| Na^+^ |  |  |  |  |  |  |  |  |  | -.458 | .351 | .392 | -.278 | -.711 |
| Mg^2+^ |  |  |  |  |  |  |  |  |  |  | **-.894*** | .271 | -.626 | **.891*** |
| Cl^-^ |  |  |  |  |  |  |  |  |  |  |  | -.603 | .594 | -.785 |
| NO_3_^-^ |  |  |  |  |  |  |  |  |  |  |  |  | -.493 | .012 |
| SO_4_^2-^ |  |  |  |  |  |  |  |  |  |  |  |  |  | -.464 |

TOC: total organic carbon; EC: electrical conductivity; TDS: total dissolved solids; ORP: Oxidation-Reduction Potential; DO: dissolved oxygen.

⁎ Indicate significant differences at *p* < 0.05. ⁎⁎ Indicate significant differences at *p* < 0.01.

**Table S4**

Pearson correlation coefficients of microbial diversity indicators and physicochemical parameters in Longkou.

| Diversity index | Temp | pH | DO | ORP | EC | TDS | TOC | K^+^ | Ca^2+^ | Na^+^ | Mg^2+^ | Cl^-^ | NO_3_^-^ | SO_4_^2-^ | HCO_3_^-^ |
| --- | --- | --- | --- | --- | --- | --- | --- | --- | --- | --- | --- | --- | --- | --- | --- |
| Chao1 | .380 | -.339 | -.190 | .273 | -.096 | -.185 | .124 | **.804**** | -.201 | .048 | -.082 | -.319 | -.264 | .454 | .106 |
| Shannon | .067 | .172 | -.506 | .347 | .023 | -.545 | .283 | .146 | **-.652*** | -.510 | -.066 | -.539 | **-.694*** | .019 | **-.633*** |
| Coverage | -.242 | .093 | -.113 | .003 | .361 | .243 | .004 | -.626 | .248 | -.002 | .117 | .297 | .127 | -.252 | -.016 |
| Heip | -.101 | .350 | -.401 | .104 | -.039 | -.506 | .319 | -.211 | -.617 | -.477 | -.103 | -.402 | **-.703*** | -.187 | **-.671*** |

TOC: total organic carbon; EC: electrical conductivity; TDS: total dissolved solids; ORP: Oxidation-Reduction Potential; DO: dissolved oxygen.

⁎ Indicate significant differences at *p* < 0.05. ⁎⁎ Indicate significant differences at *p* < 0.01.

**Table S5**

Pearson correlation coefficients of microbial diversity indicators and physicochemical parameters in Cele.

| Diversity index | Temp | pH | ORP | EC | TDS | TOC | K^+^ | Ca^2+^ | Na^+^ | Mg^2+^ | Cl^-^ | NO_3_^-^ | SO_4_^2-^ | HCO_3_^-^ |
| --- | --- | --- | --- | --- | --- | --- | --- | --- | --- | --- | --- | --- | --- | --- |
| Chao1 | .056 | **.771**** | **-.639*** | .516 | .545 | .193 | .427 | **-.633*** | .620 | .396 | .515 | -.556 | .536 | .060 |
| Shannon | .458 | **.808**** | -.536 | .317 | .347 | -.386 | .230 | **-.923**** | .423 | .183 | .353 | **-.823**** | .301 | -.446 |
| Coverage | .198 | **-.674*** | .383 | -.463 | -.484 | -.158 | -.338 | .452 | -.560 | -.419 | -.458 | .448 | -.485 | -.075 |
| Heip | .526 | .397 | -.161 | .019 | .097 | **-.646*** | .040 | **-.746*** | .132 | -.021 | .116 | -.575 | .023 | -.574 |

TOC: total organic carbon; EC: electrical conductivity; TDS: total dissolved solids; ORP: Oxidation-Reduction Potential; DO: dissolved oxygen.

⁎ Indicate significant differences at *p* < 0.05. ⁎⁎ Indicate significant differences at *p* < 0.01.

**Table S6**

Pearson correlation coefficients of microbial diversity indicators and physicochemical parameters in Wuhan.

| Diversity index | Temp | pH | DO | ORP | EC | TDS | TOC | K^+^ | Ca^2+^ | Na^+^ | Mg^2+^ | Cl^-^ | NO_3_^-^ | SO_4_^2-^ | HCO_3_^-^ |
| --- | --- | --- | --- | --- | --- | --- | --- | --- | --- | --- | --- | --- | --- | --- | --- |
| Chao1 | .316 | .191 | -.081 | .646 | .359 | .359 | -.577 | .595 | .265 | .252 | .299 | -.147 | .169 | -.122 | -.111 |
| Shannon | .653 | -.400 | **-.826*** | -.003 | **.966**** | **.966**** | .434 | **.849*** | **.947**** | -.495 | **.941**** | -.803 | .236 | -.486 | .790 |
| Coverage | .008 | -.403 | -.466 | -.550 | .355 | .355 | **.943**** | .022 | .393 | -.637 | .326 | -.364 | -.017 | -.138 | .610 |
| Heip | .418 | -.329 | -.750 | -.176 | **.857*** | **.857*** | .723 | .746 | **.852*** | -.567 | **.844*** | -.697 | .124 | -.494 | **.820*** |

TOC: total organic carbon; EC: electrical conductivity; TDS: total dissolved solids; ORP: Oxidation-Reduction Potential; DO: dissolved oxygen.

⁎ Indicate significant differences at *p* < 0.05. ⁎⁎ Indicate significant differences at *p* < 0.01.
